# Model-Free RL or Action Sequences?

**DOI:** 10.3389/fpsyg.2019.02892

**Published:** 2019-12-20

**Authors:** Adam Morris, Fiery Cushman

**Affiliations:** Department of Psychology, Harvard University, Cambridge, MA, United States

**Keywords:** reinforcement learning, action sequences, model-free control, habit, decision-making

## Abstract

The alignment of habits with model-free reinforcement learning (MF RL) is a success story for computational models of decision making, and MF RL has been applied to explain phasic dopamine responses (Schultz et al., 1997), working memory gating (O'Reilly and Frank, 2006), drug addiction (Redish, 2004), moral intuitions (Crockett, 2013; Cushman, 2013), and more. Yet, the role of MF RL has recently been challenged by an alternate model—model-based selection of chained action sequences—that produces similar behavioral and neural patterns. Here, we present two experiments that dissociate MF RL from this prominent alternative, and present unconfounded empirical support for the role of MF RL in human decision making. Our results also demonstrate that people are simultaneously using model-based selection of action sequences, thus demonstrating two distinct mechanisms of habitual control in a common experimental paradigm. These findings clarify the nature of habits and help solidify MF RL's central position in models of human behavior.

## 1. Introduction

Sometimes people make decisions by carefully considering the likely outcomes of their various options, but often they just stick with whatever worked in the past. For instance, people sometimes flexibly plan a new route to work when their old route is under construction, but sometimes they follow the old route anyway. This fundamental distinction—often cast as "planned" vs. "habitual" behavior—animates a century of decision-making research and organizes a wide array of human and non-human behaviors (Dolan and Dayan, 2013).

This distinction is commonly formalized within the “reinforcement learning” (RL) framework (Sutton and Barto, 1998; Dolan and Dayan, 2013). In this framework, planning is a form of explicit expected value maximization, or “model-based” reinforcement learning (Daw et al., 2011; Doll et al., 2015). But what is the appropriate formal description of habitual action?

**Figure 1:**
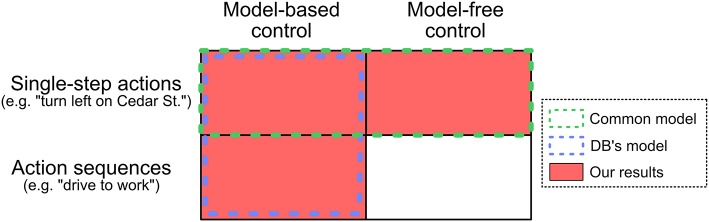
Relationship between various models. Human behavior in sequential decision making tasks is often modeled as the red squares: a mixture of model-based and model-free control of single-step actions. Dezfouli and Balleine (2012, 2013) argue that previous empirical results can be explained by the blue outline: model-based control of single-step actions and action sequences. We report evidence that people simultaneously use both model-based control of action sequences and model-free control of single-step actions.

Currently, two basic accounts compete (Figure 1). The first posits that habits arise from a representation of historical value, averaging across similar past episodes—a form of *model-free reinforcement learning* (MF RL) (Schultz et al., 1997; Glascher et al., 2010; Dolan and Dayan, 2013). In other words, people repeat actions when they have been rewarded often in the past. For instance, a person might habitually pull their smart-phone out of their pocket when standing in line because they have often enjoyed using their phone in similar past circumstances.

In contrast, the second posits that habits arise from the “chunking” of actions into sequences that often co-occur (Dezfouli and Balleine, 2012, 2013; Dezfouli et al., 2014). For instance, the sequence of actions that a person uses when tying their shoes co-occurs commonly, and so this sequence has been “chunked.” Although the chunk itself may be assigned value and controlled by an instrumental system, the elements within the chunk are not assigned value; a person executing a chunked action sequence is simply on auto-pilot.

These models are regarded as competitors because they offer divergent accounts for many of the same empirical phenomena. Most pointedly, a recent influential critique from Dezfouli and Balleine (DB) (Dezfouli and Balleine, 2012, 2013; Dezfouli et al., 2014) seeks to explain current behavioral and neural evidence for model-free RL instead in terms of action sequences selected by a superordinate planning process. In other words, they posit that model-free RL is not employed by humans; value representations are employed exclusively during model-based planning, and habitual action exclusively reflects chunked action sequences.

In theory, however, these proposed mechanisms are not incompatible—they could operate side-by-side within a single cognitive architecture. Here, we show that both model-free RL and chunked action sequences simultaneously contribute to human decisions. To do this, we modify a popular set of “two-step” behavioral tasks to isolate unique behavioral signatures of each. Using the modified tasks, we demonstrate both (a) model-based control of action sequences (consistent with DB), but (b) model-free control of single, non-sequenced actions (inconsistent with DB). Thus, our results indicate two important and distinct forms of behavioral organization that contribute to “habitual” (i.e., non-planned) action.

We first review the reinforcement learning framework, and then present the standard two-step task designed to distinguish between MF and MB influence on choice. Then, following DB, we show how (for a particular representation of the task's reward structure) model-based selection of chunked action sequences can produce seemingly MF-like behavior on this standard task. Finally, we demonstrate that an alternate variant of the task predicts separate behavioral signatures for model-free control and action sequences, and we present two experiments in which people simultaneously exhibit both signatures.

## 2. Two Models of Habitual Action

Reinforcement learning offers a powerful mathematical framework for characterizing different types of decision algorithms, and allows us to conceptually and empirically distinguish between two forms of habitual action: (1) model-free RL, and (2) model-based RL with action sequences. In this section, we first introduce the classic distinction between model-based and model-free control, and describe an experimental paradigm, the “two-step task,” which was purported to provide evidence for model-free control in humans. We then introduce DB's “action sequences” critique, and show how a model-based algorithm with action sequences could produce the patterns of habitual behavior in the original two-step task.

Before continuing, there are two theoretical issues worth clarifying. First, throughout this paper, we assume that the behavior produced by a model-free RL controller maps onto our intuitive notion of “habitual” behavior. This assumption, though common (Glascher et al., 2010; Dolan and Dayan, 2013), has been disputed (Miller et al., 2019). We do not engage with this important debate here. Our experiments dissociate model-free RL from model-based action sequences, and test whether humans actually employ model-free RL. If it turns out that model-free RL is not the right description of true habits, but instead represents a different type of unplanned behavior, then our results should be reinterpreted in that light.

Second, throughout this paper, we take “model-free” to mean a type of decision controller that does not store or use information about its environment's “transition function”—i.e., what the consequences in the environment will be of taking an action from a particular state. It is sometimes difficult to draw a sharp line between model-free and model-based algorithms; there may be a spectrum between them (Miller et al., 2019). Nonetheless, there is a clear distinction between the two ends of the spectrum, with model-free algorithms relying primarily on caching from experience with minimal prospection at decision time, and model-based algorithms relying primarily on forward planning over a model of the environment's transition function. For our simulations and model-fitting, we will rely on algorithms considered canonical examples of each type (Sutton and Barto, 1998).

### 2.1. Model-Based and Model-Free Reinforcement Learning

In the RL framework, an agent is in an environment characterized by the tuple (*S, A, T, R*), where *S* is the set of states that the agent can be in, *A* is the set of actions available at each state, *T* is a function describing the new state to which an action transitions, and *R* is a function describing the reward attained after each transition (Sutton and Barto, 1998). (For simplicity, we assume there is no discounting). The agent's goal is to find a policy—a function that describes the probability of taking each action in each state—that maximizes the agent's long-term reward. To accomplish this, the agent estimates the sum of expected future rewards following each action, called the action's "value," and then simply chooses actions with high values. We will denote the value of an action *a* in state *s* as *Q*(*s, a*).

In model-based RL, the agent learns a representation of the transition function *T*′ and reward function *R*′^1^. For instance, the agent might represent that taking action 1 in state 3 has a 40% chance of leading to state 4—or formally, *T*′(*a* = 1, *s* = 3, *s*′ = 4) = 0.4. (This is analogous to representing the consequences of one's actions—i.e., “turning left at this intersection will lead to Cedar Street”). Then, the agent might represent that transitioning to state 4 gives a reward of +10. (This is analogous to representing the desirability of those consequences—i.e., “Cedar Street is the fastest way to work”). Before making a choice, a model-based agent can recursively integrate over the decision tree implied by these two representations to compute the precise value of each available action:

(1) $$\begin{array}{l} Q_{MB}\left(s,a\right)=\underset{s^{\prime }}{\sum }T^{\prime }\left(s,a,s^{\prime }\right)*\left(R^{\prime }\left(s,a,s^{\prime }\right)+\underset{a^{\prime }\in A}{\max}Q_{MB}\left(s^{\prime },a^{\prime }\right)\right) \end{array}$$

where *s* is the agent's current state, *a* is the action under consideration, and *s*′ are the possible subsequent states.

In contrast, model-free agents do not represent the transition or reward functions—i.e., they don't prospect about the consequences of their actions. Instead, model-free agents estimate action values directly from experience, and cache these value representations so they can be accessed quickly at decision time. (In other words, instead of learning that “turning left at this intersection will lead to Cedar street”, a model-free agent will have simply learned that “turning left at this intersection is good”). In the popular model-free algorithm Q-learning (Watkins and Dayan, 1992), for instance, action values are updated *after* each choice according to the following formula:

(2) $$\begin{array}{l} Q_{MF}\left(s,a\right)\leftarrow Q_{MF}\left(s,a\right)+\alpha *\left(r+\underset{a^{\prime }\in A}{\max}Q_{MF}\left(s^{\prime },a^{\prime }\right)-Q_{MF}\left(s,a\right)\right) \end{array}$$

where *s* is the agent's current state, *a* is the chosen action, *s*′ is the subsequent state, *r* is the reward received, and α is a free parameter controlling the learning rate. By incorporating both the immediate reward and the next state's action values into the update rule, the *Q*(*s, a*) value estimates converge to the long-term expected reward following each action, and Q-learning agents learn to maximize long-term reward accumulation without explicitly representing the consequences of their choices.

[Note that, although canonically considered a model-free algorithm (Sutton and Barto, 1998), Q-learning involves some minimal type of prospection: It uses value estimates of the actions *a*′ in the subsequent state *s*′ to update its value estimate for selecting *a* in *s*^2^. As discussed above, the line between model-free and model-based is not always sharp (Miller et al., 2019). Nonetheless, like standard model-free algorithms, Q-learning does not use an explicit model of the transition function *T*. Moreover, its lack of forward planning at decision time means that it produces the standard signature of model-free control in the task used here, which we describe below. Hence, it is an appropriate formalization of model-free RL for our purposes].

Model-free RL is a particularly powerful model of habitual behavior. It captures human and animal behavior in a variety of paradigms (Glascher et al., 2010; Daw et al., 2011; Dolan and Dayan, 2013), as well as behavioral deficits in obsessive-compulsive disorder (Voon et al., 2015), Parkinson's (Frank et al., 2004), and drug addiction (Redish, 2004). It elegantly explains phasic dopamine responses in primate midbrain neurons (Schultz et al., 1997) and BOLD signal changes in the human striatum (Glascher et al., 2010). Finally, it forms the basis of models of other cognitive processes, such as moral judgment (Crockett, 2013; Cushman, 2013), working memory gating (O'Reilly and Frank, 2006), goal selection (Cushman and Morris, 2015), and norm compliance (Morris and Cushman, 2018).

### 2.2. The Two-Step Task

**Figure 2:**
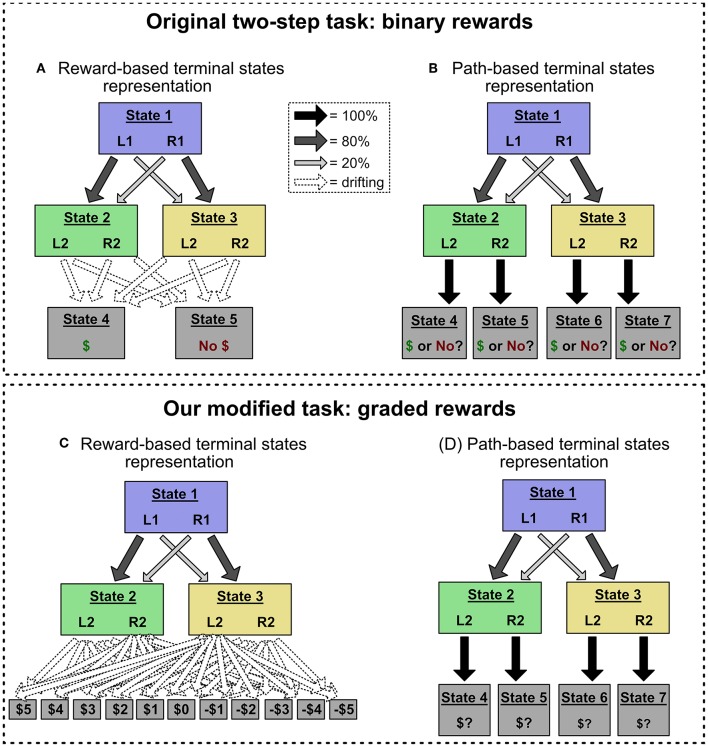
**(A,B)** The original two-step task, which uses binary reward outcomes. This task is often represented with the structure in **(A)**, in which terminal states are defined by their reward values (e.g., State 4 gives a reward of 1, State 5 gives a reward of 0), and drifting reward probabilities are encoded as transition probabilities to those terminal states. However, the task can also be represented with the structure in **(B)**, in which each Stage 2 choice leads to a unique terminal state (choosing L2 in State 2 leads to State 4, and so on). In this alternate representation, drifting reward values are encoded as the value of those “path-based” terminal states. We show that action sequences can only mimic model-free choice patterns in the reward-based terminal state representation. **(C,D)** Our modified task, which uses graded reward outcomes (i.e., –5 through 5). Using graded reward outcomes precludes the reward-based terminal state representation, which would require eleven terminal states and forty-four transition probabilities (shown in **C**). Instead, this modified task induces the alternate, path-based terminal state representation (shown in **D**), allowing us to deconfound action sequences and model-free control.

The difference between model-based and model-free control can be illustrated in a popular sequential decision paradigm called the "two-step task" (Daw et al., 2011). In the two-step task, participants go through a series of trials and make choices that sometimes lead to reward. On each trial, they make two choices (Figure 2A). The first choice (“Stage 1”) presents two options (“Left” and “Right”) that we label L1 and R1. These actions bias probabilistic transitions to two subsequent states (“Stage 2” states) which are yellow and green, and which are not rewarded. For instance, L1 might typically lead to a green screen, and R1 to a yellow screen. After transitioning to one of the Stage 2 states, people then make a second choice between two further options, L2 and R2. These each probabilistically transition to one of two terminal states: a state with reward, or a state without reward. These transition probabilities drift over the course of the experiment. Thus, to maximize earnings, participants must continually infer which Stage 2 state-action pair has the highest probability of reward, and make choices in both stages to attain that outcome.

The two-step task was initially designed to distinguish between model-based and model-free control of single-step, non-sequenced actions. The key logic of this experimental design depends on the probabilistic transitions between Stage 1 and Stage 2 (Figure 2A). 80% of the time, L1 leads to green and R1 to yellow. But, 20% of the time, the transitions are reversed. Participants' choices following rare transition trials reveal the distinction between model-free and model-based RL. Imagine an agent chooses L1, gets a rare transition to yellow, chooses R2, and receives a reward. How will that reward affect behavior on the next trial? A model-based agent will, using its internal model of the task, increase its value estimate of the Stage 1 action that typically leads to yellow: R1. [Formally, in Equation 1, the value of *Q*(*State*3, *R*2) will get applied primarily to *Q*(*State*1, *R*1), not *Q*(*State*1, *L*1), because the former has a higher probability of transitioning to State 3]. In contrast, a model-free agent, who has not represented the transition structure, will increase its value estimate of the Stage 1 action it chose: L1. In other words, a model-based agent's response to reward or no reward will depend on whether the preceding transition was rare or common; but a model-free agent will respond by becoming more or less likely to repeat its last choice, no matter the transition type.

This logic leads to clear behavioral predictions. If an agent is model-based, the probability of repeating a choice will depend on the interaction between the reward type (reward vs. no reward) and the transition type (common vs. rare). In contrast, if an agent is model-free, the probability of repeating a choice will depend on the reward type only. When humans play the two-step task, they consistently show a mixture of both approaches (Glascher et al., 2010; Daw et al., 2011). They show both an interaction between reward and transition type (signature of MB RL), and a main effect of reward (signature of MF RL). The interpretation is that people are sometimes planners (captured by MB RL) and sometimes habitual (captured by MF RL). This finding is a pillar of support for the case that humans employ model-free RL in decision making.

### 2.3. The Action Sequences Critique

However, as DB show, the behavioral pattern in the original two-step task can be explained without invoking model-free RL. Instead, DB argue, people are employing model-based selection of chained action sequences (Dezfouli and Balleine, 2013). An action sequence is a series of actions that are precompiled into a single representation. For instance, a person tying her shoelaces does not consider each step in the sequence separately; rather, she simply chooses the abstract option "tie my shoes," and then executes the sequence of lower-level actions automatically. Similarly, a person driving to work may not consider each turn to be a new decision. Rather, she made only one decision, in which she chose the option "drive to work"; and the sequence of lower-level actions (e.g., start the car, turn left onto Cedar Street) followed automatically. Crucially, the action sequence model posits that the internal structure of the option is not guided by a value function; this is the key point of divergence with standard MF RL methods.

It is uncontroversial that people employ action sequences in some form (Dezfouli and Balleine, 2012, 2013; Dezfouli et al., 2014). We do not detail all the evidence DB marshal for the existence of action sequences. Rather, we focus on one key hypothesis: that action sequences can fully explain away any apparent role of MF RL in human behavior. Specifically, we focus on the claim that action sequences can produce the standard signature of MF RL in the two-step task.

#### 2.3.1. Action Sequences in the Two-Step Task

**Figure 3:**
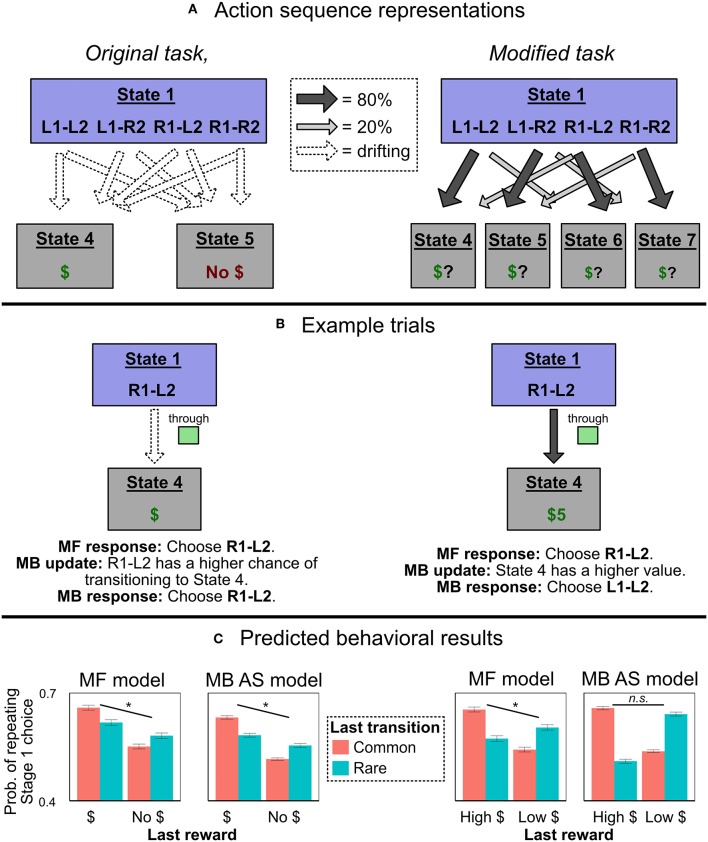
Experiment 1 predictions. **(A)** How an agent using action sequences would represent the two task variants. In the original task with binary outcomes, they are represented as actions which lead directly, with drifting probabilities, to one of two reward states. In the modified task, they are represented as actions which lead, with fixed probabilities, to different terminal states with drifting values. **(B)** Example trials in which an agent chooses the action sequence R1-L2, receives a reward, and updates its beliefs. In the original task representation, both MF and MB controllers have the same response, allowing the model-based action sequences model to produce MF-like behavior; in the alternate representation, the MF and MB responses diverge. **(C)** Simulated probability of Stage 1 choice in the two representations, as a function of last trial's reward and transition type. We compared a traditional, flat model with partial MF control (“MF model”) to an action sequences model with only MB control (“MB AS model”). The action sequence model produces MF-like behavior in the original representation, but not the alternate one. (Asterisks and “n.s.” refer to the significance of the main effect of reward in each simulation. Error bars are ±1 SEM).

On the action sequences model, when people make a Stage 1 choice, they employ model-based RL to choose between six possible options: the two single-step actions L1 and R1, and four action sequences L1-L2, L1-R2, R2-L1, and R2-L2 (Figure 3A). If a person chooses a single-step action like L1, she transitions to either the green screen or yellow screen and then uses that information to make her Stage 2 choice. But if a person chooses an action sequence like L1-L2, she selects L1 and then L2, no matter what screen she transitions to. In other words, she employs a form of “open-loop control” that is insensitive to information obtained during execution of the action sequence (Dezfouli and Balleine, 2012).

To see how the introduction of action sequences could explain seemingly model-free behavior in the two-step task, imagine that a participant chooses L1-L2, passes through yellow (rare transition), and receives a reward. Importantly, “receiving a reward” in the original paradigm is indicated by transitioning to a screen with a picture of money on it. This “rewarded” terminal state is not in any way specific to the path the person took to it—every unique sequence of actions terminates in one of two identical states: one that is rewarded, and another that is not. Thus, an agent who was insensitive to information obtained during the action sequence could learn from the reinforcement experience without ever referencing whether it had transitioned to the yellow or green state. All she would learn is that she had chosen the sequence L1-L2, and ended up at the screen with reward. (In figurative terms, when exiting “autopilot,” she would know if she got money, but not where she had been). If rewarded, then, on the next trial, when consulting her internal model of the environment, she would become more likely to stay with L1-L2, not switch to R1-L2 (left-hand-side of Figure 3B). In this way, a purely model-based agent could mimic the signature of MF algorithms, and human behavior on the original two-step task can be explained without reference to MF RL.

In this paper, we demonstrate that action sequences can only produce MF-like behavior for this particular reward structure with binary outcomes. Then, in two experiments, we modify the two-step task to induce an alternate reward structure in which action sequences cannot produce MF-like behavior, and show that people still exhibit the behavioral signatures of MF RL. At the same time, our paradigm also produces unambiguous evidence that people do employ model-based control of action sequences. We conclude that people's habit-like behavior can be produced by both model-free RL and action sequences.

## 3. Simulations: Action Sequences Can Only Mimic MF-Like Behavior for a Particular Reward Structure

Although not previously emphasized, the action sequences model can only produce MF-like behavior in the original two-step task because the task has a peculiar property: The terminal reward conditions can plausibly be represented as two unified reward states (one for a reward, one for no reward), subject to drifting transition probabilities from each Stage 2 state-action pair (e.g., green-L2, yellow-R2). In other words, for any given action sequence that is selected at the beginning of the task, “reward probability” and “state transition probability” coincide perfectly—the relevant states are simply *defined* in terms of reward. For example, suppose an agent selects and executes the action sequence L1-L2, and that she then receives a reward. The result is encoded as an increased probability of L1-L2 transitioning to the “reward” state (i.e., State 4 in Figure 3A). Or, if she instead chooses R1-L2 and receives a reward, the result is again encoded as an increased probability of R1-L2 transitioning to the “reward” state. This representational scheme has an important consequence: Model-based selection of action sequences is insensitive to the distinction between common and rare transitions.

Consider, however, an alternative representation of the reward structure (Figure 3B). Here, the current expected reward from each Stage 2 state-action pair is incorporated into the value of a separate terminal state. For example, if the participant chooses R1-L2, passes through green, and receives a reward, she increases the value of the terminal state associated with green-L2 (State 4). Crucially, under this alternate task representation, model-based selection of action sequences cannot produce MF-like behavior. The critical test is: After choosing a sequence like R1-L2, passing through green, and receiving a reward, will she increase the probability of choosing R1-L2 (the MF-like option) or L1-L2 (the MB-like option)? Under the alternate representational scheme, a model-based planner will recognize that L1-L2 is more likely than R1-L2 to lead to the high-reward terminal state green-L2 (right-hand-side of Figure 3C). Thus, a model-based planner will not show the signature of model-free control, and cannot explain MF-like behavior in this version of the two-step task.

Put differently, in order for model-free and model-based controllers to make different behavioral predictions after a rare transition, the model-based controller needs to incorporate the fact that it was a rare transition into its post-trial update. When it chooses an action sequence, remains on autopilot through Stage 2, and arrives at an undifferentiated terminal state (the original task representation), the fact that it experienced a rare transition is not represented (explicitly or implicitly). But in the alternate task representation, the fact of the rare transition is encoded into the terminal state itself and, thus, it is naturally encoded in the MB controller's post-trial update.

In sum: The two-step task was designed to produce divergent behavior for model-free and model-based controllers after a rare transition. DB showed that, in the original task, a model-based controller with action sequences predicts the MF-like behavioral response (repeating the same Stage 1 choice after a rewarded rare transition). We show that this is only true for a “reward-based terminal state” representation of the task; in a “path-based terminal state” representation of the task, a MB controller with action sequences returns to predicting the MB-like, not MF-like, response^3^. We now report simulations confirming this theoretical analysis.

### 3.1. Methods

We simulated two algorithms: one that employed a weighted mixture of model-based and model-free control (the “MF model”), and one that employed only model-based control but included action sequences (the “MB AS model”). In both algorithms, model-based and model-free Q-values were computed as described in section II; model-based Q-values^4^ were computed by recursively applying Equation (1), while model-free Q-values were computed via Q-learning (Equation 2). For the model-free Q-values, we included eligibility traces, with decay parameter λ. This means that, after participants chose an action in Stage 2, the reward prediction error was immediately “passed back” to update the Stage 1 action (discounted by λ; see Sutton and Barto, 1998). (The presence of eligibility traces are critical for the analysis of the two-step task described above. Without eligibility traces, a reward on trial *t* would not immediately influence Stage 1 choice on trial *t* + 1; see Daw et al., 2011).

#### 3.1.1. MF Model

In the MF model, agents estimate both model-based and model-free Q-values for single-step actions; these estimates must be integrated to ultimately produce a choice. How RL agents should, and how people do, arbitrate between model-based and model-free systems is a complex and important topic (Daw et al., 2005; Kool et al., 2017; Miller et al., 2019). Here, following past work (e.g., Daw et al., 2011; Cushman and Morris, 2015), we sidestep this question and assume that the model-based and model-free Q-values are ultimately combined with a mixture weight ω:

$$\begin{array}{l} Q_{combined}\left(s,a\right)=\omega *Q_{MB}\left(s,a\right)+\left(1-\omega \right)*Q_{MF}\left(s,a\right) \end{array}$$

ω = 1 leads to pure model-based control, and ω = 0 leads to pure model-free control. This formalization is agnostic between different interpretations of the actual integration process, such as agents alternating between model-based and model-free systems on different trials, or agents estimating both types of Q-values on each trial and weighting them together. For a discussion of the distribution of ω values observed in our experiments, see the trial-level model fitting sections below. (For an in-depth analysis of the arbitration problem, see Kool et al., 2017).

After combining the model-based and model-free Q-values, agents chose actions with probability proportional to the exponent of the combined Q-values (plus a “stay bonus” capturing the tendency to repeat previous actions^5^). Formally, the probability of choosing action *a* in state *s* was given by a softmax function with inverse temperature parameter β, with a stay bonus ν:

$$\begin{array}{l} Prob\left(s,a\right)=\frac{e^{\beta *Q_{combined}\left(s,a\right)+\nu *1_{a=a_{prev}}}}{\sum_{a^{\prime }\in A}e^{\beta *Q_{combined}\left(s,a^{\prime }\right)+\nu *1_{a^{\prime }=a_{prev}}}} \end{array}$$

We used separate inverse temperature parameters for Stage 1 and Stage 2 choices. The MF model did not include action sequences.

#### 3.1.2. MB AS Model

The MB AS model differed from the MF model in two ways. First, it employed only model-based Q-values to select actions (i.e., ω = 1). Second, it included action sequences. In the MB AS model, in addition to being able to choose the two single-step actions in Stage 1, agents could also choose four additional action sequences: L1-L2, L1-R2, R1-L2, and R1-R2. Agents chose between all these options via a softmax function over the model-based Q-values (with a stay bonus). If the agent chose an action sequence in Stage 1, it executed the Stage 2 action automatically; if it chose a single-step action, then, at Stage 2, it made a second choice between the two single-step actions L2 and R2.

When using action sequences, there is a question of when to update their value estimates: Should an agent update its value estimate of an action sequence only after having selected it *as an action sequence*, or additionally after having chosen the single-step actions that happen to correspond to the sequence? Concretely, after choosing the single-step actions L1 and R2 (without invoking action-sequence control), should the agent then update the value representation associated with the action sequence L1-R2? Following Dezfouli and Balleine (2013), we present results assuming that the agent does update sequences after choosing their component actions; this assumption probably better captures how sequences are “crystallized” in real life (Dezfouli and Balleine, 2012; Dezfouli et al., 2014). However, all our results are similar if we assume the agent does not.

#### 3.1.3. Parameter Values

Both models had a learning rate, two inverse temperatures, and a stay bonus. We used the same parameter distributions as Dezfouli and Balleine (2013). For each agent, the learning rate was randomly sampled from *Beta*(1.1, 1.1); the inverse temperatures from *Gamma*(1.2, 5); and the stay bonus from *Normal*(0, 1). The MF model had two additional parameters: the mixture weight ω, which was sampled from *Uniform*(0, 1), and the eligibility trace decay parameter λ, which was also sampled from *Uniform*(0, 1).

We simulated 1,000 agents of each type playing each task variant (one with a reward-based terminal state representation, and one with a path-based terminal state representation). All agents performed 125 trials.

#### 3.1.4. Analysis

Following the logic in section 2, we tested whether each model produced the signature of model-free control by estimating a logistic mixed effects models, regressing a dummy variable of whether they repeated their Stage 1 choice on (a) the last trial's transition type (common vs. rare), (b) the last trial's reward, and (c) their interaction. The classic signature of model-free control in this setting is a main effect of last trial's reward on Stage 1 choice.

### 3.2. Results

In the original task representation, both algorithms showed a main effect of reward on Stage 1 choice (left-hand-side of Figure 3C; for MF model, *p* < 0.0001; for MB AS model, *p* < 0.0001). But in the alternate representation, only the algorithm with model-free control showed a main effect of reward (right-hand-side of Figure 3C; for MF model, *p* < 0.0001; for MB AS model, *p* = 0.57).

### 3.3. Discussion

We simulated two algorithms—one that included model-free RL, and one that was purely model-based but included action sequences—and found the result predicted by our analysis. In the original task with a reward-based terminal state representation, model-based control of action sequences can mimic the signature of model-free control; but with a path-based terminal state representation, model-based control of action sequences cannot mimic model-free control. Thus, if people continue to show MF-like behavior in a version of the two-step task that induces the alternative representation, it would demonstrate that action sequences cannot account for all MF-like behavior.

In this simulation, we only reported the patterns of Stage 1 choices. Following past work (Daw et al., 2011; Cushman and Morris, 2015; Gillan et al., 2016), Stage 1 choice is the key variable we use to test for an effect of model-free control, and hence was the focus of this simulation. However, testing for an effect of model-free control is not our only goal; we also hope to show that people are simultaneously using action sequences (in Experiment 1), and to test whether those action sequences are themselves under model-free or model-based control (in Experiment 2). For those purposes, we will end up relying on two other outcome variables: Stage 2 choices, and the reaction times of Stage 2 choices. If people are employing action sequences, then their Stage 2 choices and reaction times will exhibit a unique pattern noted by Dezfouli and Balleine (2013) and described below in Experiment 1. Hence, Stage 2 choice and RT will be used in Experiment 1 to test for the presence of action sequences. Moreover, in the simulations for Experiment 2, we will show that Stage 2 choice and reaction time can also be used to distinguish between model-free and model-based control of action sequences. This logic will be described in section 5.

## 4. Experiment 1: Disambiguating Control With a Graded Reward Structure

In our first experiment, we adopt a modified version of the two-step task that induces the “path-based terminal state” representation. In the original version, the amount of reward present in each terminal state was constant (e.g., 1 bonus point), and what drifted throughout the task was each Stage 2 state-action pair's probability of transitioning to the reward state vs. the non-rewarded state. For example, green-L2 might initially have a 75% chance of giving 1 bonus point, but later it might only have a 25% chance. This configuration supported the representation in Figures 2A, 3A, where drifting rewards are encoded as transitions probabilities to terminal states associated with “reward” or “no reward.”

In our version, rewards could take on a range of point values, and what drifted was the number of points associated with each Stage 2 state-action pair (Kool et al., 2016). For example, green-L2 might initially be worth 3 points, but later it might be worth -4 points. (Point values were restricted to [–5, 5] and drifted via a reflecting normal random walk with μ = 0, σ = 1.75). This configuration induces the “path-based terminal state” representation in Figure 2D. To see why, imagine a person trying to use the original “reward-based terminal state” representation in our modified task. The person would have to represent eleven separate terminal states (one for each possible point value), and forty-four terminal transition probabilities (Figure 2C). This would be a very inefficient representation and so we consider it unlikely. We further encouraged the path-based terminal state representation by reformatting the reward screen to clearly indicate which Stage 2 state-action pair had been chosen.

Thus, in this modified task, we assume that participants represent the task with path-based terminal states, and thus this task deconfounds the signatures of action sequences and model-free control. If, in this task, people still exhibit the behavioral signature of model-free control—a main effect of reward on subsequent Stage 1 choice—then it cannot be explained by model-based selection of action sequences.

Of course, graded rewards are not a new innovation, and have been used in several past studies (Cushman and Morris, 2015; Kool et al., 2016). Our contribution is to leverage graded rewards to deconfound the behavioral signatures of action sequences and MF RL. We collected new data, rather than reanalyze past studies, to ensure that the details of the task design were appropriate for the present question.

### 4.1. Methods

One hundred and one participants were recruited on Amazon Mechanical Turk. (We blocked duplicate IP addresses, only allowed IP addresses from the United States, and only used workers who had done over 100 previous studies on Turk with an overall approval rating of at least 95%). All participants gave informed consent, and the study was approved by Harvard's Committee on the Use of Human Subjects.

We used the version of the two-step task described in Kool et al. (2017), which has a cover story about spaceships to make the task more understandable. We explained the task in detail to participants, including explicitly telling them the transition structure. After being explained the task, participants completed 25 untimed practice trials which did not count toward their bonus payment. After the practice trials, participants were given a review of the task. Finally, they completed 125 real trials, in which each choice had a 2 s time limit.

Following Dezfouli and Balleine (2013), we did not counterbalance which side of the screen the actions appeared on; L1 was always on the left, R1 on the right, and so on. This feature maximizes the potential for participants to employ action sequences.

Participants were excluded if they completed the instructions in less than 1 min (suggesting that they did not read carefully); although the experiment was not pre-registered, this exclusion criterion was chosen in advance. Five participants were excluded, leaving 96 for the analyses.

We analyzed people's Stage 1 choices using logistic mixed effects models, regressing a dummy variable of whether they repeated their Stage 1 choice on (a) the last trial's transition type (common vs. rare), (b) the last trial's reward, and (c) their interaction. We included all random intercepts and slopes, and computed *p*-values with Wald z-tests. We estimated correlations between random effects, except in models with three-way interaction terms (where we disallowed random effect correlations to support model convergence). We report unstandardized regression coefficients as *b*. (We analyzed people's Stage 2 choices similarly).

### 4.2. Results

#### 4.2.1. Signature of Model-Free RL

**Figure 4:**
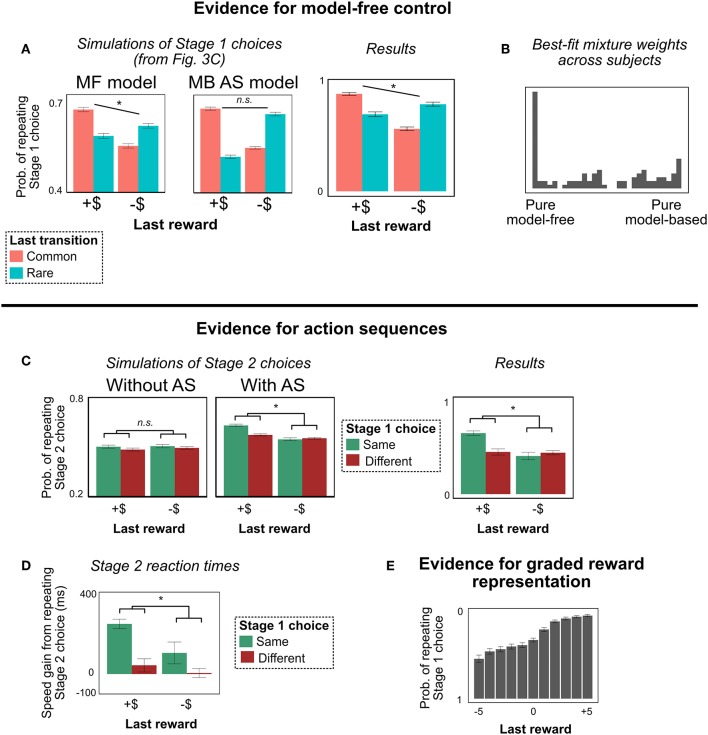
Experiment 1 results. (For graphical convenience, we bin “last reward” into two categories: positive (“+$”) and negative (“–$”); we do not mean to imply that people actually represent the rewards this way. All statistical analyses are computed with unbinned reward). **(A)** Stage 1 choices as a function of last trial's reward and transition type. People showed MF-like behavior (main effect of reward), even under the alternate task representation. **(B)** Histogram of best-fit mixture weights ω across subjects. People showed substantial model-free control. **(C)** Stage 2 choices as a function of last trial's reward and this trial's Stage 1 choice (on trials with a rare transition, following trials with a common transition). People's decisions to repeat their Stage 2 choices were more correlated with their decisions to repeat their Stage 1 choices following a reward—a unique behavioral signature of action sequences (Dezfouli and Balleine, 2013). **(D)** Stage 2 reaction times. People are faster to repeat their Stage 2 action, and this effect is strongest for trials following a reward where they repeated their Stage 1 action. This pattern is another signature of action sequences. **(E)** Probability of repeating Stage 1 choice as a function of last trial's unbinned reward. People are sensitive to the graded nature of the rewards, suggesting that they are not binning them into “positive”/“negative” categories. (This result is important for ensuring that people are employing a path-based, not reward-based, representation of the terminal states). All error bars are ±1 SEM; asterisks indicate the significance of the main effect of last trial's reward (in **A**), or the interaction between last trial's reward and this trial's Stage 1 choice (in **C**,**D**).

The results of Experiment 1 are shown in Figure 4. People continue to show the signature pattern of MF-like behavior (Figure 4A). For Stage 1 choice, in addition to the interaction between last reward and transition type (signature of MB RL; *b* = 0.29, *z* = 12.4, *p* < 0.0001), there was a main effect of last reward (signature of MF RL; *b* = 0.16, *z* = 8.4, *p* < 0.0001). This result provides an example of MF-like behavior that cannot be explained by action sequences, and is the key finding of Experiment 1.

#### 4.2.2. Concurrent Evidence for Action Sequences

Although action sequences cannot explain MF-like behavior in our task, we did find concurrent evidence that people are employing action sequences in this paradigm. This evidence is important, because it suggests that our task alteration did not discourage people from using action sequences; rather, people seem to employ MF RL and action sequences simultaneously. (We will also exploit these behavioral signatures in Experiment 2 in order to test for model-based vs. model-free control of action sequences).

The first piece of evidence for action sequences derives from logic originally presented by DB (Dezfouli and Balleine, 2013). Consider a trial in which a person chooses an action sequence, experiences a common transition, and receives a reward or punishment. She should be more likely to repeat the sequence on the following trial if she receives a reward, as opposed to a punishment^6^. Moreover, a consequence of her tendency to repeat the action sequence is that her decisions to repeat Stage 1 and Stage 2 actions will be correlated: If she repeats the same Stage 1 action, she will be more likely to repeat the same Stage 2 action. Putting these ideas together, a signature of action sequences is that repetition of Stage 1 and repetition of Stage 2 actions will be more correlated following a reward than following a punishment (simulations in Figure 4C).

This signature, however, is insufficient. There is an alternate explanation for it: Following a reward, a person using single-step actions should be more likely to repeat the same actions on the next trial. Thus, any factors which make her more likely to repeat her Stage 1 action—e.g., she was paying more attention on that trial—would make her more likely to repeat her Stage 2 action also, inducing a correlation.

To remedy this issue, we follow DB and restrict our analysis to trials in which the current Stage 2 state is different from the previous Stage 2 state (Dezfouli and Balleine, 2013). For instance, on the last trial, a person may have chosen R1-L2 and gone through the yellow state to State 6; but on this trial, the person may have chosen R1-L2 and, due to a rare transition, gone through the green state to State 4. If the correlation between Stage 1 and Stage 2 actions is due to action sequences, this restriction won't matter; people executing an action sequence are on “autopilot,” and won't alter their behavior based on the Stage 2 state. But if the correlation is due to confounding factors like attention, then this restriction should eliminate the effect: A reward on the last trial would not influence a single-step agent's choice in a different Stage 2 state^7^.

As in prior work (Dezfouli and Balleine, 2013), people in our task showed precisely this pattern (interaction *b* = 0.11, *z* = 7.4, *p* < 0.0001), suggesting that they are indeed using action sequences (Figure 4C). However, the results in section 4.2.1 indicate that they are not relying on pure model-based control; there is a model-free influence on their choice. Hence, it appears that people are using both types of unplanned choice mechanisms: model-free control and action sequences. (For a discussion of how often people use each mechanism, see the section on trial-level model fitting below).

**Figure A1:**
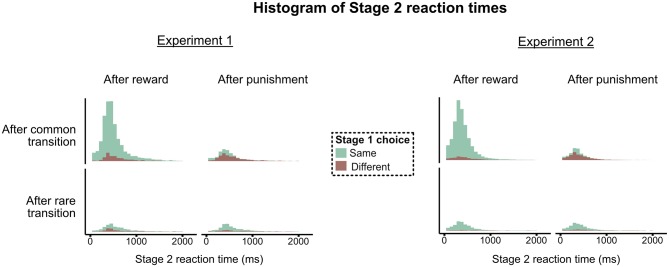
Distribution of Stage 2 reaction times, as a function of the previous trial's transition and reinforcement and the current trial's Stage 1 choice. All distributions were roughly unimodal and right-skewed.

An additional signature of action sequences appears in participant's Stage 2 reaction times. While executing a sequence, people don't have to make any further decisions (e.g., to compare the values of alternative actions), and hence should be faster at selecting actions. This fact, combined with the effect above, leads to the following prediction. As described above, people tend to repeat the same sequence on consecutive trials following a reward. This implies that, on trials where they repeat their Stage 1 action, people should be faster to select a response in Stage 2 if they are repeating the same Stage 2 choice—i.e., if they are following the prescription of the action sequence. (We again restrict this analysis to trials following a common transition, with a different Stage 2 state than the previous trial). To test this prediction, we computed the difference in reaction times between trials when people repeated their Stage 2 action and trials when they didn't, conditioning on (a) whether they received a reward or punishment last trial, and (b) whether they repeated their Stage 1 choice. Replicating Dezfouli and Balleine (2013), we find the predicted interaction: People are faster when repeating their Stage 2 action, and this effect is strongest on trials following a reward where people chose the same Stage 1 action (*b* = 14.9, *t* = 5.2, *p* < 0.0001)^8^. The interaction is key: The fact that the boost in reaction time is stronger when participants chose the same Stage 1 action, and when they received a reward on the last trial, suggests that the effect is not due to an inherent time cost of switching Stage 2 actions (which would produce a main effect where people are always faster to choose the same Stage 2 action). The interaction is a unique signature of action sequences (Dezfouli and Balleine, 2013). (The raw reaction time data are presented in Figure A1 in Appendix).

#### 4.2.3. Are People Representing the Rewards as Graded?

As discussed above, the logic of Experiment 1 depends on people using the path-based, not reward-based, terminal state representation (Figure 2D, not Figure 2C). The reward-based terminal state approach is an implausible representation of a task with graded rewards. However, it is possible that, even though the task *has* graded rewards, people are not representing it that way; they could be representing the rewards as binary (e.g., either positive or negative). This possibility is problematic for our analysis, because it means that people could still be using the reward-based terminal state representation.

To demonstrate that people are treating the rewards as graded, we examined Stage 1 choices after rewards of each point value. (In this analysis, we focus exclusively on trials following common transitions). The results are shown in Figure 4E. People are clearly representing the full range of rewards, and not just binning them into positive or negative—every increase in point value is associated with an increase in stay probability. We tested this statistically by comparing two logistic mixed effects models: one that predicted Stage 1 choice from last trial's graded reward (i.e., the actual point value), and one that predicted choice from last trial's binned reward (i.e., either positive or negative). The former was heavily preferred (AIC of the former model was over 458 less than the AIC of the latter). Thus, the reward-based terminal state approach remains an implausible representation of our task^9^.

### 4.3. Trial-Level Model Fitting

As an additional analysis, we fit several variants of the model-free and action sequence models to participant choices at a trial level, and used Bayesian model selection to adjudicate between them. We fit five models: one model that did not use sequences, and four models that used sequences and employed different elements of MF and MB control (Table 1). For each model, we first estimated each subject's maximum *a posteriori* parameters, using the same priors as in the simulations and the *fmincon* function in MATLAB. To get each subject's best-fit parameters, we reran the optimization procedure ten times with randomly chosen parameter start values and selected the overall best-fitting values. We then used the Laplace approximation to compute the marginal likelihood for each subject for each model (Daw, 2011), and used the random-effects procedure of Rigoux et al. (2014) to estimate protected exceedance probabilities (PXPs)—i.e., the probability that each model is the most prevalent in the population.

**Table 1:** Models used in model comparison, and comparison results.

**Model**	**Uses sequences**	**Control of single-step actions**	**Control of action sequences**	**PXP in Expt. 1**	**PXP in Expt. 2**
No sequences	No	Mixture of MF and MB	N/A	0.40	0
Pure MB	Yes	MB	MB	0	0
Mixture-actions/MB-sequences	Yes	Mixture of MF and MB	MB	0	1
MB-actions/Mixture-sequences	Yes	MB	Mixture of MF and MB	0	0
Mixture-actions/Mixture-sequences	Yes	Mixture of MF and MB	Mixture of MF and MB	0.60	0

*PXP stands for protected exceedance probabilities (Rigoux et al., 2014)*.

*In Experiment 1, the preferred model used a mixture of model-free and model-based methods to evaluate both single-step actions and action sequences (although it was closely followed by a model that omitted action sequences entirely). In Experiment 2, the preferred model used a mixture of model-free and model-based methods to evaluate single-step actions, but only model-based methods to evaluate action sequences – consistent with the behavioral results of Experiment 2*.

The results are shown in Table 1. The preferred model used a mixture of model-free and model-based values to select both single-step actions and action sequences (PXP = 0.60), although it was closely followed by the model that did not use action sequences (but still used a mixture of MF and MB values to select single-step actions; PXP = 0.40). Analyzing the subject-level mixture weight ω, we find that subjects' behavior showed substantial model-free influence (Figure 4B); the mean weight was 0.44, and the distribution was peaked near 0 (pure model-free), with only 16% of subjects showing a weight greater than 0.9.

These results are consistent with our central claim that people are employing model-free control in this task. On the other hand, these results are mixed about whether people are using action sequences. Given the strong behavioral evidence in favor of action sequences, both in our experiments and past work (Dezfouli and Balleine, 2013), we think it likely that most subjects were using them; this inconsistency in the model-fitting suggests that the behavioral results may provide more reliable tests of our hypotheses (see Palminteri et al., 2017 for an in-depth argument in favor of this approach). Nonetheless, we include the model-fitting results here for completeness. We consider inconsistencies between the model-fitting and behavioral results in the section 6.

One question that model-fitting can help answer is how often people employ each choice mechanism. The mean mixture weight was 0.44. This number could mean different things depending on the interpretation of action selection. If people are employing model-free methods on some trials and model-based methods on others, then a mean ω of 0.44 indicates that people would on average be employing model-free RL on 56% of trials. If people are instead averaging model-free and model-based values together on each trial, then a mean ω of 0.44 indicates that model-free value makes up 56% of the final value estimate. We remain agnostic between these interpretations (Kool et al., 2017). Either way, there was substantial between-subject variation in mixture weights (Figure 4B).

A more difficult question to answer is on what percentage of trials people are using action sequences. We do not know when a person used an action sequence, nor it is a parameter explicitly estimated in the model-fitting procedure (Dezfouli and Balleine, 2013). We leave this question to future research.

### 4.4. Discussion

We modified the two-step task to induce an alternate reward representation in which model-based selection of action sequences could not produce MF-like behavior. In this modified task, people still showed the same behavioral pattern, including the signature main effect of reward on Stage 1 choice. This analysis suggests that people are employing MF RL in some capacity—even in a task where they are also using action sequences. Here, action sequences and MF RL seem to be complements, not competitors.

One potential concern with this experiment is that people are using a different representation of the task than the one we assume (Figure 2D). We believe it is implausible that people are using the reward-based terminal state representation assumed by Dezfouli and Balleine (2013) (Figure 2C); however, there is another representation that could be problematic for our analysis. Specifically, people might be collapsing States 4–7 into one undifferentiated terminal state, with the rewards encoded into the preceding actions—e.g., the reward in State 4 might actually be encoded as the reward from choosing L2 in State 2, with the terminal state ignored. This representation would be problematic for our analysis because a model-based action sequence controller could plausibly, after exiting the action sequence, be aware of the reward it received without being aware of the path it took to get that reward. Hence, a model-based action sequence controller could ignore the transition type, and mimic model-free behavior^10^.

A similar worry goes as follows. Even if people are using our assumed path-based terminal state representation (Figure 2D), a MB controller could still in principle select some type of “extended” action sequence that ignores the identity of the terminal state. For instance, imagine a MB controller chooses L1-L2, gets a rare transition in the middle of the sequence, receives a reward, and ignores the associated terminal state (e.g., State 6) because it is still on “autopilot.” This model-based controller would credit the reward to the sequence L1-L2 itself, and not to the and would thus appear model-free. This concern makes it seem as if a model-based controller can still mimic a model-free one in our task.

*A priori*, there is some reason to doubt these concerns. We clearly differentiated the four terminal states with unique visual features, which included an image of the last action taken to reach that terminal state. Moreover, people could not quickly pass through the screen indicating the terminal state; they were required to remain on that screen for several seconds. If an action sequence controller is ignoring all this easily-accessible information about the transition structure and instead crediting the reward directly to the action sequence itself, then it is not obvious that the controller is still model-based. It is showing no sensitivity to the task's transition structure, and instead caching value directly to actions themselves—the definition of a model-free controller.

Nonetheless, we seek direct evidence against this possibility. Experiment 1 tested for the presence of model-free control, but it was not designed to test which type of controller was being used to select action sequences specifically. In the next experiment, we modify the design to produce a unique behavioral signature of model-free and model-based control of action sequences. This design allows us to address the aforementioned concern in the following way. If people are using an “unresponsive” model-based controller to select action sequences in a way that ignores the identity of the terminal state and mimics model-free control (e.g., through an undifferentiated terminal state representation, or an “extended” sequence), then we should find evidence of apparent model-*free* control of action sequences. Conversely, if we find no evidence of apparent model-free control of action sequences, that result would suggest that people do not use an “unresponsive” model-based controller for this family of tasks—and hence that the MF-like behavior in Experiment 1 was not produced by such a controller, and was genuinely model-free^11^.

To preview our results, we find that people are *not* exhibiting apparent model-free control of action sequences; they instead produce the behavioral signature of accurate model-based control of action sequences (with knowledge of the differentiated terminal states). Yet, they still exhibit a signature of some type of model-free control. Together, this pattern suggests that people are not exhibiting apparent model-free control via an unresponsive model-based action sequence controller; rather, they are exhibiting genuine model-free control of single-step actions.

## 5. Experiment 2: Testing for Model-Based vs. Model-Free Control of Action Sequences

Experiment 2 was designed to answer the question: Which type of controller is being used to select action sequences? In principle, MB and MF control can be applied to both single-step actions and action sequences (Figure 1). Models of choice in the two-step task commonly assume that single-step actions are controlled by a mixture of model-based and model-free control (green box in Figure 1; Glascher et al., 2010; Daw et al., 2011; Kool et al., 2017). But what about action sequences? DB posited that action sequences would be chosen exclusively by MB control, but their paradigm did not allow them to test this claim. By using graded rewards in a modified task structure, we can test for unique behavioral signatures of model-based and model-free control of action sequences. We find strong evidence that, as DB predicted, action sequences are under model-based control. In contrast, although we find clear evidence that people are employing *some* type of model-free control, we find no evidence that they are using model-free RL to select action sequences. This result helps address the concern from Experiment 1—that an unresponsive model-based action sequence controller was mimicking model-free control—by simultaneously demonstrating (a) model-free control of some type, but (b) no apparent model-free control of action sequences. More broadly, this result suggests that two types of habitual mechanisms coexist in this paradigm: (accurate) model-based selection of action sequences, and model-free control of single-step actions.

### 5.1. Logic of Experiment 2

**Figure 5:**
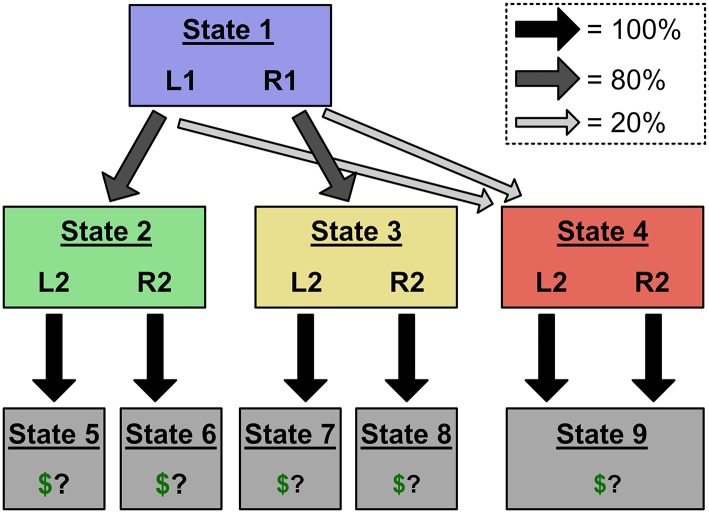
Design of Experiment 2. Rare transitions now lead to a common red state with a single reward outcome. This prevents experience in the red state from affecting a MB controller's decisions, allowing us to isolate MF behavior.

The second experiment differed from the first only in the transition structure between Stages 1 and 2 (Figure 5). As before, L1 and R1 had an 80% chance of transitioning to the green and yellow states, respectively. But in Experiment 2, both actions have a 20% chance of transitioning to a novel red state (State 4). Since both Stage 1 actions have the same chance of transitioning to the red state, the value of State 9 should not influence a model-based controller's choices in Stage 1; a model-based controller will integrate out any experience it has in the red state, and be unaffected by feedback from State 9. This fact, combined with the effect of action sequences on Stage 2 choices and reaction times described in Expt. 1, elicits unique behavioral predictions for model-based and model-free selection of action sequences.

**Figure 6:**
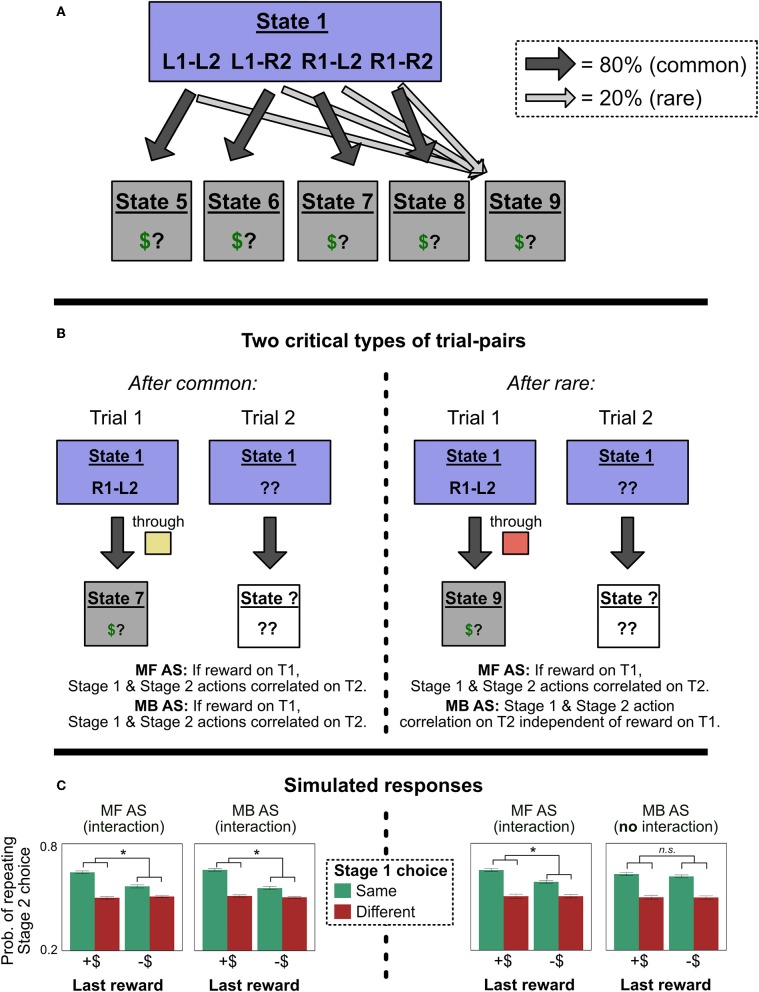
Predictions in Experiment 2. **(A)** The representation of Experiment 2 by an agent using action sequences. All sequences have an 80% chance of leading to their respective terminal states, and a 20% chance of leading to State 9. This design ensures that a model-based controller's decisions will not be influenced by the value of State 9. **(B)** Two types of critical trials. On the left, we analyze trials following common transitions. Here, both model-free and model-based action sequence models (MF AS and MB AS) predict an interaction between Stage 1 choice and last trial's reward on Stage 2 choice. On the right, we analyze trials following rare transitions. Here, MF AS predicts the same interaction, but MB AS predicts that the interaction should disappear, because the value of State 9 will not matter for sequence selection. (This analysis is restricted to instances in which Trial 2 has a different Stage 2 state than Trial 1; this restriction rules out confounds described in Experiment 1). **(C)** Simulations to confirm the predictions in **(B)**. All error bars are ±1 SEM; asterisks indicate significant interactions.

The key to Experiment 2 is that, following trials with transitions to the red state, a person using model-free control to select action sequences will show the Stage 2 action sequence effects, while a person using model-based control to select sequences will not (Figure 6A; right-hand-side of Figure 6B). Recall from Experiment 1 that, if a person is using action sequences, their Stage 2 choices will be predicted by a positive two-way interaction between last trial's reward and this trial's Stage 1 choice: They will be more likely to repeat their Stage 2 choice after being rewarded last trial and repeating their Stage 1 choice this trial (Figure 4C). [As described above, this interaction occurs because people will be most likely to be repeating an action sequence, and hence to repeat their Stage 2 choice, following reward and repeated Stage 1 choice. The same is true for their reaction times: They will be *fastest* to repeat their Stage 2 choice after being rewarded last trial and repeating their Stage 1 choice this trial. See Figure 4D. As in Experiment 1, we rule out confounds by restricting this analysis to trials in which the Stage 2 state differs from the previous trial, which does not matter for an action sequence controller because it is insensitive to transitions while executing the sequence (Dezfouli and Balleine, 2013)]. Experiment 2 combines this fact with a design ensuring that only a model-free controller will be affected by reinforcement after a rare transition; a model-based controller will ignore the reinforcement (Figure 5). In Experiment 2, if people are using model-free control of action sequences, they will show the signature of action sequences (the two-way interaction of last trial's reward and this trial's Stage 1 choice on Stage 2 choice/reaction time) after a rare transition; but if they are using model-based control of action sequences, they won't show this signature. (Both controllers will show the signature after common transitions; left-hand-side of Figure 6B). Hence, if people exhibit this two-way interaction after both common and rare transitions, we can infer that their action sequences are under some degree of model-free control. In contrast, if people exhibit this two-way interaction in common but not rare transitions, we can infer that their action sequences are under model-based control. (And if people exhibit the interaction after neither type of trial, we would infer that they are not using action sequences at all). These effects are summarized in Table 2.

**Table 2:** Key predictions in Experiment 2.

**Sequence controller**	**Predicted pattern after common transitions**	**Predicted pattern after rare transitions**
Pure MF, or mixture of MF/MB	2-way interactions of S2 choice ~ S1 choice * Last reward, S2 RT drop ~ S1 choice * Last reward	2-way interaction of S2 choice ~ S1 choice * Last reward, S2 RT drop ~ S1 choice * Last reward
Pure MB	2-way interaction of S2 choice ~ S1 choice * Last reward, S2 RT drop ~ S1 choice * Last reward	No 2-way interactions
No sequences	No 2-way interactions	No 2-way interactions

S1 stands for Stage 1; S2 stands for Stage 2; S2 RT drop indicates the predicted gain in speed (and hence drop in reaction time) from repeating a Stage 2 choice. If a person is using model-free control of action sequences, they will show the action sequences' signature two-way interactions (Stage 2 choice/RT drop ~ Stage 1 choice * Last trial's reward) after both a common and rare transition. But if a person is using model-based control of action sequences, they will show the interactions after common but not rare transitions, and hence will show three-way interactions of Stage 2 choice/RT drop ~ Stage 1 choice * Last reward * Last transition type.

Note that, if people are using model-based control of action sequences, we can go one step further in our analysis. As just discussed, we predict that in this case people would show the two-way interaction after common transitions but not rare transitions. Statistically, this means that they would show a significant two-way interaction after common transitions, a null effect for the interaction after rare transitions—and, critically, a significant *three*-way interaction when including common vs. rare transition as an additional regressor. In other words, they will show a significantly stronger interaction after common transitions than after rare transitions. This result would provide positive evidence for model-based control of action sequences that goes beyond a null effect after rare transitions.

To preview our results, we find precisely the patterns predicted by model-based control of action sequences: People show the signature two-way interaction (in both Stage 2 choices and reaction times) after common transitions but not rare transitions, and show a three-way interaction when including transition type as a regressor. This is strong evidence that, at the higher level of the action hierarchy (i.e., action sequences), people in this paradigm employ model-based control. At the same time, we find concurrent evidence that people are employing model-free control at some point in their decision making. Hence, our results again suggest that model-free control and action sequences coexist in people's decision making process, and that, at least in this paradigm, model-free control may be more strongly applied at lower levels of the action hierarchy.

### 5.2. Simulations

We confirm this analysis by simulating agents performing the task in Experiment 2 (Figure 6C). We used the same methods as in the prior simulations, with one change. The two algorithms now both used a mixture of model-free and model-based Q-values to assign value to single-step actions (e.g., L1, R1), and both employed action sequences (e.g., L2-R2); they differed only in the type of value assignment to action sequences. One algorithm used model-free Q-values to assign value to action sequences (“MF AS”), while the other algorithm used model-based Q-values (“MB AS”).

The results confirmed our theoretical analysis. After trials with a common transition, both MF AS and MB AS agents showed the predicted two-way interaction: Their Stage 2 choices were predicted by their Stage 1 choices times last trial's reward (*p*′*s* < 0.0001; left-hand-side of Figure 6C). In contrast, after trials with a rare transition, only MF AS agents showed the two-way interaction (*p* < 0.0001); MB AS agents showed no interaction (*p* = 0.71; Bayes factor in favor of null is 88; right-hand-side of Figure 6C). Moreover, when including last trial's transition type as a regressor, MB AS agents showed the predicted three-way interaction (*p* < 0.0001). These simulation results confirm the theoretical analysis above, and demonstrate that this paradigm can detect unique effects of model-free and model-based action sequence control. Next, we test for these effects empirically.

### 5.3. Methods

Three hundred participants were recruited on Amazon Mechanical Turk, using the same filtering criteria as in Experiment 1. The task was identical to Experiment 1, except for the change in the state/transition structure. We excluded 18 participants who finished the instructions in less than 1 min, and 1 participant for whom the study severely glitched.

In the instructions, we emphasized to people that the transition probabilities to the rare state did not change over the course of the experiment, and that when a rare transition happened was completely random with no way to plan for it. To ensure that participants believed this key part of the experimental design, we added a question at the end of the experiment: “Did you believe that, on any given round, the two Stage 1 choices had the same probability of transitioning to the red state?” (We also added a second question: “Did you believe that, on any given round, the two actions in the red state always led to the same amount of bonus money?” The significance of this belief is discussed below). We excluded an additional 84 participants who answered “No” to either of these questions, leaving 197 participants for analysis. Also, at the end of the instructions, we included three comprehension check questions, asking, for each Stage 2 state, which of the Stage 1 actions was most likely to reach it (or whether both actions were equally likely). Participants generally understood the transition structure: The percentage of participants giving correct answers for the three Stage 2 states were (in order): 87, 94, and 95%. If participants got the comprehension check question wrong, they were told the correct answer and reminded of the transition structure (but not excluded). Again, although these results were not pre-registered, all exclusion criteria were chosen in advance.

As can be seen in Figure 5, both actions in the red state lead to the same outcome; participants were told this fact explicitly. This design feature ensured that all action sequences had the same probability of transitioning to State 9, and that a model-based controller would not incorporate information from rare-transition trials into its subsequent Stage 1 choice.

All statistical methods are similar to those in Experiment 1. Bayes factors were computed with a BIC approximation (Wagenmakers, 2007).

### 5.4. Results

#### 5.4.1. Evidence for Model-Free RL

**Figure 7:**
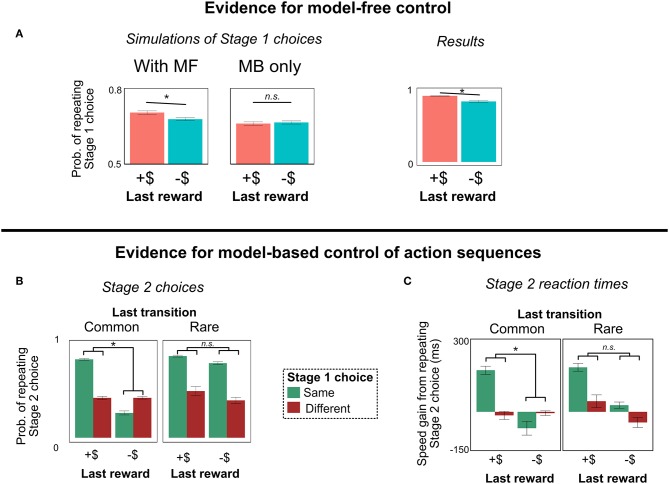
Results of Experiment 2. **(A)** Evidence for model-free control. After trials with a rare transition, people are more likely to repeat their Stage 1 choice if rewarded; purely model-based agents (with or without action sequences) do not show this pattern. **(B,C)** Evidence for model-based control of action sequences. People show the action sequence signature—a tendency to repeat their Stage 2 choice most often following a reward on the last trial and a repeat of their Stage 1 choice on this trial (i.e., the interaction Stage 2 choice ~ Stage 1 choice * Last reward)—following a common transition, but not a rare transition. Their reaction times show a similar pattern. These patterns are predicted by model-based, but not model-free, control of action sequences. All error bars are ±1 SEM; asterisks indicate a significant main effect of last trial's reward (in **A**) or significant interactions between last trial's reward and this trial's Stage 1 choice (in **B**,**C**).

First, we conceptually replicate the finding from Experiment 1 that model-free RL influences choice. In this paradigm, the signature of MF RL is simple (Cushman and Morris, 2015). If people are using MF RL, their Stage 1 choice should be influenced by the reward received on a rare transition; they should be more likely to repeat their Stage 1 choice after a reward in State 9, compared to a punishment. But if they are using only model-based RL (with or without action sequences), their Stage 1 choice should not be influenced by the value of State 9 (simulations in Figure 7A).

Indeed, people show the signature of MF RL (Figure 7A). They are more likely to repeat their Stage 1 choice following a more positive reinforcement in State 9 (main effect of last reward; *b* = 0.20, *z* = 7.0, *p* < 0.0001). This result demonstrates that MF RL influences people's choice in some way in this paradigm.

#### 5.4.2. Evidence for Model-Based Selection of Action Sequences

Second, we turn to the main question of Experiment 2: Does model-free RL influence people's choices of action sequences, or are action sequences controlled primarily by model-based RL?

In this paradigm, people seem to choose action-sequences primarily through model-based RL (Figure 7B). As predicted by MB RL, in trials following a rare transition, there is no interaction on Stage 2 choice between Stage 1 choice and last reward (interaction term, *b* = 0.022, *z* = 1.1, *p* = 0.27, *BF*_*null*_ = 41). This result suggests that people are not using model-free RL to select action sequences.

Moreover, we find positive evidence for model-based control of sequences. Regressing people's Stage 2 choices on (a) their Stage 1 choice, (b) last trial's reward, and (c) last trial's transition type, we find the predicted three-way interaction: People show the signature of action sequences [an interaction between (a) and (b)] more in trials following a common transition, compared to trials following a rare transition (Figure 7B; interaction term, *b* = 0.44, *z* = 10.7, *p* < 0.0001). This is precisely the pattern predicted by model-based control of action sequences.

A similar signature of model-based control of action sequences comes from people's reaction times in Stage 2. As described above, if people are using model-based control of action sequences, then their gain in speed from repeating their Stage 2 choice should be predicted by the two-way interaction of their Stage 1 choice and last trial's reward—but only following common, not rare, transitions. And indeed, people exhibit precisely this pattern (Figure 7C): They showed the predicted interaction after common transitions (*b* = 48.7, *t* = 10.8, *p* < 0.0001), no interaction after rare transitions (*b* = −7.1, *t* = −1.1, *p* = 0.26—although the Bayes factor was weak, *BF*_*null*_ = 2.5), and a significant interaction between those two effects when including transition type as a regressor (*b* = 55.2, *t* = 6.8, *p* < 0.0001)^12^.

### 5.5. Trial-Level Model-Fitting

As an additional analysis, we fit the same models from Experiment 1 to trial-level choices in Experiment 2 (using identical procedures as before). The preferred model used a mixture of model-free and model-based methods to evaluate single-step actions, but only model-based methods to evaluate action sequences (*PXP* = 0.999). This result is consistent with the behavioral results in Experiment 2. (On the other hand, it is inconsistent with the model-fitting result in Experiment 1. We return to this issue in section 6).

### 5.6. Discussion

We replicated the finding from Experiment 1 that people are employing model-free control of some sort. Moreover, we found evidence that people's choice of action sequences was under model-based, and not model-free, control. People's patterns of Stage 2 choice qualitatively matched the simulated behavior of agents using model-based control of action sequences. Additionally, the best-fitting model used a mixture of model-free and model-based control to select single-step actions, but only model-based control to select action sequences.

These results validate the hypothesis of Dezfouli and Balleine (2013) that action sequences would be under model-based control. On the other hand, they further reinforce our primary claim that model-free RL is part of people's decision making repertoire, and not explained away by model-based control of action sequences. In particular, they provided evidence against the concern raised at the end of Experiment 1: that people could be using an unresponsive model-based action sequence controller which mimics model-free control. If that were the case, we would have seen evidence of apparent model-free control of action sequences in Experiment 2. Instead, we find that people select action sequences using an accurate model-based method, but select single-step actions with some degree of model-free control.

We do not make the strong claim that people never exhibit unresponsive model-based control of action sequences, or genuine model-free control of action sequences. It is difficult to draw strong conclusions from a null result. Nonetheless, in our paradigm, model-free control appears to be applied primarily to lower levels of the action hierarchy. We return to this question in section 6.

One worry with this experiment is that it depends on people believing that the two Stage 1 actions had the same probability of transitioning to the rare state on each trial. If people were committing a “hot hands” fallacy and believing that a Stage 1 action that produced a rare transition last trial was more likely to produce one this trial, that mistaken belief could potentially produce apparent model-free behavior (Gilovich et al., 1985). We mitigated this risk by repeatedly emphasizing to people that the transition probabilities did not change from trial to trial, and that each rare transition was unpredictable and independent of the others. Moreover, we excluded participants who reported not believing this fact. Nonetheless, it is possible that this belief persisted in polluting our data. Future work should rule out this potential confound more thoroughly.

## 6. General Discussion

Our work aligns with many prior studies arguing that some form of model-free RL is implemented by humans. Model-free RL has proved a successful model of human and animal behavior in sequential decision tasks (Dolan and Dayan, 2013), phasic dopamine responses in primate basal ganglia (Schultz et al., 1997), fMRI patterns during decision making (Glascher et al., 2010), and more. We defend this model against a recent critique (Dezfouli and Balleine, 2012, 2013; Dezfouli et al., 2014) by providing unconfounded evidence that, in a variant of the popular two-step task, people *do* employ model-free RL, and not just model-based control of chained action sequences.

At the same time, our work provides strong evidence that, in addition to model-free RL, people indeed employ model-based control over action sequences. This result suggests that the puzzle of habits will not be solved by one model; “habits” likely comprise multiple decision strategies, including both model-free RL and action sequences.

### 6.1. Relationship Between Behavioral and Model-Fitting Results

We presented two types of evidence: one-trial-back behavioral effects (e.g., the effect of last trial's reinforcement on this trial's choice), and model-fitting results. In general, these methods were in agreement. In Experiment 1, both methods indicated that people were employing model-free RL and (generally) action sequences. In Experiment 2, both methods indicated that people were using model-free RL to evaluate single-step actions, but only model-based RL to evaluate action sequences. This concordance reinforces those claims.

There were, however, two points on which the model-fitting results were inconsistent. First, in Experiment 1, the model-fitting suggested that many of the participants were not actually using action sequences. This is possible, but seems unlikely in light of our clear behavioral results and the results of past work (Dezfouli and Balleine, 2012, 2013; Dezfouli et al., 2014). Second, the preferred model differed between Experiments 1 and 2. In Experiment 2, the preferred model used only model-based RL to evaluate action sequences, but in Experiment 1 the preferred model used both model-based and model-free RL to evaluate them. It is possible that participants in Experiment 1 were actually using more model-free control of action sequences than in Experiment 2. On the other hand, since it was Experiment 2 that was designed to test for the type of action sequence controller, the preferred model in Experiment 2 is probably more informative on this point. In any case, the inconsistency casts doubt on the reliability of the model-fitting approach for answering these questions. We believe that our clear patterns of qualitative behavioral results are stronger evidence for our claims than the model-fitting results; for a detailed discussion of this point, see Palminteri et al. (2017).

### 6.2. Could MF-Like Behavior Be Produced by Model-Based Algorithms With Inaccurate Beliefs?

**Figure A2:**
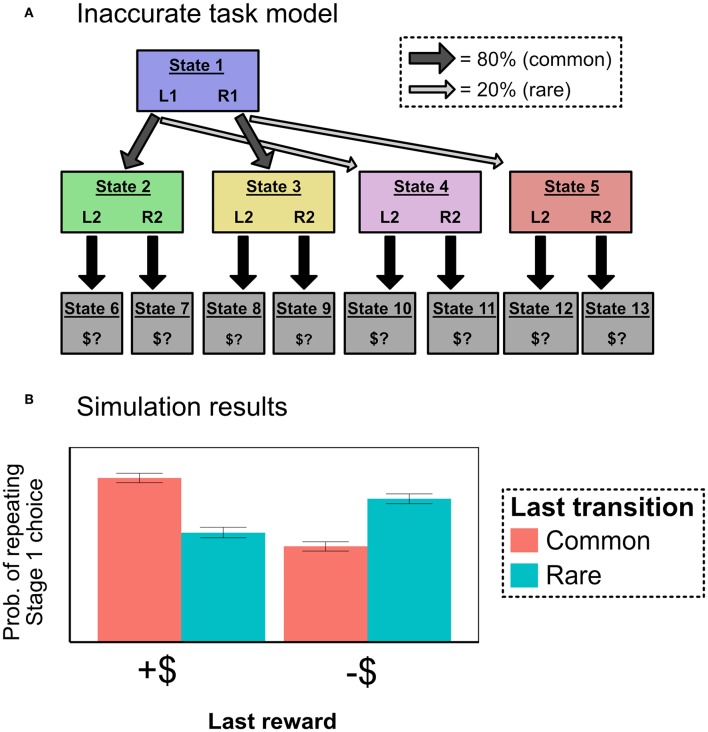
Simulations illustrating how a model-based algorithm with an inaccurate task model could mimic model-free control. **(A)** The example inaccurate task model. The algorithm believes that rare transitions lead to unique Stage 2 states. **(B)** Simulation results for agents using a mixture of an accurate model-based algorithm and the inaccurate model-based algorithm. These agents produce both an interaction between last trial's reward and transition type (the classic signature of model-based control), and a main effect of last trial's reward (the classic signature of model-free control). Hence, these agents produce MF-like behavior without MF control. We consider possibilities like these in section 6. (We thank David Melnikoff for suggesting this example).

We presented evidence for model-free control in human behavior that is deconfounded from one potential alternative: model-based control of action sequences. There are, however, other model-based algorithms that could mimic model-free control by having an inaccurate model of the task. For instance, consider a person in Experiment 1 who believes that rare transitions lead to unique Stage 2 states—e.g., that a rare transition from L1 leads to a different state than a common transition from R1 (Figure A2A in the Appendix). A model-based agent with this task model would produce MF-like behavior because it would be more likely to repeat its Stage 1 choice following both common and rare transitions (since a reward from a rare transition no longer suggests that the agent should switch its Stage 1 choice; Figure A2B). Other examples of inaccurate models that can produce MF-like behavior are given by da Silva and Hare (2019). In the most extreme case, MF-like behavior in this task an always be mimicked by an algorithm that ignores the task instructions and builds a transition model of the form “repeating behavior after being rewarded leads to more money at the end of the experiment.”

There is some reason to doubt that MF-like behavior can be explained this way, as an “inaccurate model-based” controller. A key feature of model-free RL is its computational simplicity (relative to model-based RL). This feature helps makes sense of why people would exhibit MF-like behavior relatively more when under cognitive load (Otto et al., 2013), or when the financial stakes are lower (Kool et al., 2017). These results are more difficult to explain under an “inaccurate-model-based” account, since it is not clear that using an inaccurate model of the task is more computationally efficient. Moreover, there is strong neural evidence for model-free RL that is difficult to explain under an inaccurate-model-based account (Schultz et al., 1997; Dolan and Dayan, 2013).

However, this is an active area of debate (da Silva and Hare, 2019). Here, we do not rule out all the inaccurate-model-based alternative accounts of our behavioral results, or provide definitive evidence for model-free RL. We instead make the more modest claim that the signature of model-free RL observed here is not due to model-based control of action sequences.

### 6.3. At What Level of Abstraction Does Model-Free RL Operate?

Our results contribute to an ongoing investigation into the scope of model-free RL. Model-free RL—and habits in general—are often characterized as applying to relatively concrete actions (e.g., a rat pulling a lever, or a human pushing a button). But some research has suggested that MF RL can also apply to relatively abstract “actions”, like goal selection (Cushman and Morris, 2015) or working memory gating (O'Reilly and Frank, 2006).

Here, we tackled the question of whether MF RL also applies to the control of another type of abstract action: action sequences. We found no evidence that people used model-free RL for action sequences. Rather, in Experiment 2, we found strong evidence that people used model-based RL to evaluate sequences. This result aligns with the predictions of Dezfouli and Balleine (2013) that action sequences would be under model-based control.

There are two reasons, however, not to draw strong conclusions from this result. First, it is a null result; it is possible that in other paradigms, or other experimental settings, people would have shown evidence of model-free sequence selection. Second, it is highly likely that *some* action sequences can be under model-free control. After all, the actions “pull a lever” or “push a button” actually comprise many motor subroutines—so if MF RL can apply to them, it must apply to sequences of some kind.

Nonetheless, our results raise important questions about when and how MF RL operates at higher levels of abstraction in the action hierarchy. This question is ripe for future research.

## 7. Conclusion

Humans exhibit many habit-like patterns of behavior. Our studies demonstrate one such pattern that is best explained by model-free RL, and another that is best explained by model-based selection of action sequences. This suggests that action sequences should be viewed as complements, not alternatives, to MF RL, and that combining MF RL with other approaches will give us a fuller understanding of habits.

## Data Availability Statement

All data and code used for this paper can be found at https://github.com/adammmorris/action_sequences.

## Ethics Statement

This study was approved by the Committee on the Use of Human Subjects at Harvard University under protocol IRB14-2016: A computational approach to human moral judgment. We obtained written informed consent from all participants by electronic approval at the outset of our online testing procedure.

## Author Contributions

This research was conceived and designed by AM and FC, implemented and analyzed by AM, and written by AM with assistance from FC.

### Conflict of Interest

The authors declare that the research was conducted in the absence of any commercial or financial relationships that could be construed as a potential conflict of interest.

## Footnotes

^1^In many cases, the reward function is static and given to the RL agent ahead of time. But, in our experiments (and many others; e.g., Glascher et al., 2010; Kool et al., 2017), the reward function is constantly changing, and so the agent must continually learn it.

^2^We thank a reviewer for raising this point.

^3^How does a path-based terminal state representation relate to the “Markov” assumption in reinforcement learning? Informally, the Markov assumption is that, after conditioning on the current state, the future is independent of the path taken to reach the current state. It is a key assumption in RL (Sutton and Barto, 1998). Path-based representations will still have the Markov property; if necessary, they can just build the path taken to reach a state into the representation of the state itself (e.g., if I sprint to my friend's house, the resulting state representation might include, not just “at my friend's house,” but also “exhausted from the sprint”). This kind of augmented state representation is often necessary for complex applications of reinforcement learning (Sutton and Barto, 1998). However, in the tasks we use to induce the path-based representation, that kind of augmented state representation is actually not needed, because the different terminal states (States 4–7, Figure 2) are clearly differentiated from each other. Hence, the representation for State 4 does not need to explicitly include the information “I chose L1 and L2 to get here,” because State 4 already clearly differs from the other terminal states. (Keeping with our analogy, the terminal states are more akin to different friends' houses). We still refer to this as a “path-based” terminal state representation only to emphasize that, unlike in the reward-based representation, the terminal states resulting from each Stage 2 choice path are different.

^4^To compute the model-based Q-values, agents need an estimate of the transition function. Since participants in the experiments were explicitly told the transition probabilities and given practice with them, we assumed that participants would begin the task with an accurate estimate of the transition function. Thus, we gave agents an accurate model of *T*. The results do not change if we model agents as learning the transition probabilities dynamically.

^5^Note that, although we don't give it much attention here, some recent work theorizes that the stay bonus is actually a formalization of habits that is closer to our intuitive notion of what it means to be “habitual” (Miller et al., 2019).

^6^This analysis is restricted to trials following a common transition, to avoid questions about model-based vs. model-free control of action sequences; that question is addressed in Experiment 2. Here, the goal is to demonstrate that people are using action sequences in some form.

^7^Moreover, if people are generalizing across Stage 2 states (i.e., blending together their value estimates for L2 in State 2 and in State 3), then they should exhibit a main effect of reward on Stage 2 choice—not the predicted interaction between reward and Stage 1 choice. See Dezfouli and Balleine (2013) for a more thorough justification for this test.

^8^For simplicity, we graph these results as a *two*-way interaction on the *difference* in reaction time. But, for the analysis, we properly test for a *three*-way interaction on the *raw* reaction times between last trial's reward, this trial's Stage 1 choice, and this trial's Stage 2 choice. Following DB, we did not apply a log transformation to RTs.

^9^Of course, when graphing our results, we treat the rewards as binary (positive or negative). But this approach is just for graphical convenience, and does not imply that people are representing the rewards that way.

^10^We thank a reviewer for raising this point.

^11^Though the model-fitting results in Experiment 1 suggested that people were showing apparent model-free control of action sequences, Experiment 1 was not designed to address this question, and we prefer to address the question with clear behavioral predictions (Palminteri et al., 2017). We review and consider inconsistencies in the model-fitting results in section 6.

^12^As in Experiment 1, for readability, we describe and graph the RT effects with “Stage 2 RT drop from repeating Stage 2 choice” as the dependent variable. But in our actual analysis, we test the effects with raw Stage 2 RT as the dependent variable, and Stage 2 choice as an additional regressor interacting with the others. Hence, if people are using model-based control of action sequences, they will properly show a *three*-way interaction of Stage 2 RT ~ Stage 1 choice * Last reward * Stage 2 choice after common but not rare transitions, and hence a *four*-way interaction of Stage 2 RT ~ Stage 1 choice * Last reward * Stage 2 choice * Last transition type. These interactions are what we report here.
